# VM Capacity-Aware Scheduling within Budget Constraints in IaaS Clouds

**DOI:** 10.1371/journal.pone.0160456

**Published:** 2016-08-08

**Authors:** Vasileios Thanasias, Choonhwa Lee, Muhammad Hanif, Eunsam Kim, Sumi Helal

**Affiliations:** 1 Division of Computer Science and Engineering, Hanyang University, Seoul, Republic of Korea; 2 Department of Computer Engineering, Hongik University, Seoul, Republic of Korea; 3 CISE Department, Univ. of Florida, Gainesville, FL 32611, United States of America; University of Texas at San Antonio, UNITED STATES

## Abstract

Recently, cloud computing has drawn significant attention from both industry and academia, bringing unprecedented changes to computing and information technology. The infrastructure-as-a-Service (IaaS) model offers new abilities such as the elastic provisioning and relinquishing of computing resources in response to workload fluctuations. However, because the demand for resources dynamically changes over time, the provisioning of resources in a way that a given budget is efficiently utilized while maintaining a sufficing performance remains a key challenge. This paper addresses the problem of task scheduling and resource provisioning for a set of tasks running on IaaS clouds; it presents novel provisioning and scheduling algorithms capable of executing tasks within a given budget, while minimizing the slowdown due to the budget constraint. Our simulation study demonstrates a substantial reduction up to 70% in the overall task slowdown rate by the proposed algorithms.

## Introduction

A cloud computing system offers an inexhaustible supply of resources, which can be dynamically claimed and released [1]. With cloud computing, users are not required to plan beforehand for provisioning and enterprises can start with small capital expenditures, increasing their computing resources only when a rise in the service demand occurs. The cloud system contains three major levels. a) Workflow application level is the depiction of the development and management called SaaS. b). Middleware level is the representation of workflow management enactment service known as PaaS. c). Infrastructure level epitomises the unified resources and is commonly known as IaaS. It is defined as the capability provided to the service consumer to provision the fundamental computing resources. Accordingly, IaaS enables the service consumer to deploy and execute the arbitrary software, including OS and applications [2]. Elasticity is a key aspect of cloud computing that refers to the capability to rapidly provision computing resources as needed. It can also be defined as the ability to quickly request, receive, and later release as many resources as needed [3]. By enabling to scale up or down in response to the fluctuation of resource needs, we can effectively reduce performance degradation due to system under-provisioning and the unnecessary cost of over-provisioned resources.

In recent years, there is rising interest about data analysis in scientific computing as essentially every field is witnessing an exponential growth in the volume of the data inundation. The volume of dataset points toward parallelism as being obligatory for processing the information in a timely manner. Different scientific applications such as biomedical, bioinformatics application, including PhyloD [4], Express Sequence Tag [5] (EST), and Alu [6], need this kind of parallelism to achieve their main goal of providing the results in timely fashion while analyzing large volume of datasets. These datasets are usually vulnerable and need proper security mechanism and encryption [7][8][9][10].

These large-scale cloud applications are typically hosted on a set of server instances to handle a large volume of user requests. The question is how many instances are necessary. Auto scaling is a mechanism which acquires and releases resources automatically, as the application goes through ups and downs in the demand [11]. An auto-scaling mechanism may determine not only the amount of leased resources but also the type of the resource instances. Some jobs perform better on a compute-optimized virtual machine, and others on a memory-optimized instance or on a GPU-optimized instance. One of the features missing from today’s auto-scaling mechanisms in use is the intelligence to support users’ optimization goals, such as minimizing the cost/delay or meeting budget constraint. Existing scheduling algorithms aim to increase the speed of job executions as much as possible without considering the amount of money that users are willing to spend for a job. Additionally, most works assume environments where all VMs are identical and thus all the tasks assigned to them have similar performance.

In this paper, we address the problem of executing a cloud job with a minimal delay, under a given budget constraint. We propose a budget-aware provisioning mechanism to determine the amount of resource allocations based on the remaining budget. In particular, we emphasize the efficient scheduling of jobs with heterogeneity in VM capacity. In order to evaluate our proposed schemes, we implemented a cloud workflow simulator based on CloudSim toolkit [12]. It was demonstrated that, through a set of representative simulation cases, our algorithms are cost-beneficial and capable of executing work-flows within a defined budget. Additionally, the scheduling performance can further be improved by provisioning appropriate VMs that match the types of workload tasks.

The rest of the paper is organized as follows: Section 2 describes a cloud provisioning framework and formalizes the problem we tackle. Section 3 and 4 proposes two scheduling algorithms: based and enhanced versions. Section 5 presents our efforts to evaluate the performance of our proposal. Previous works are discussed in Section 6. Finally, Section 7 concludes the paper.

## Cloud Provisioning Framework

We first bring up the problem of minimizing task delay within budget constraints for jobs submitted to the cloud.

**Fig 1:**
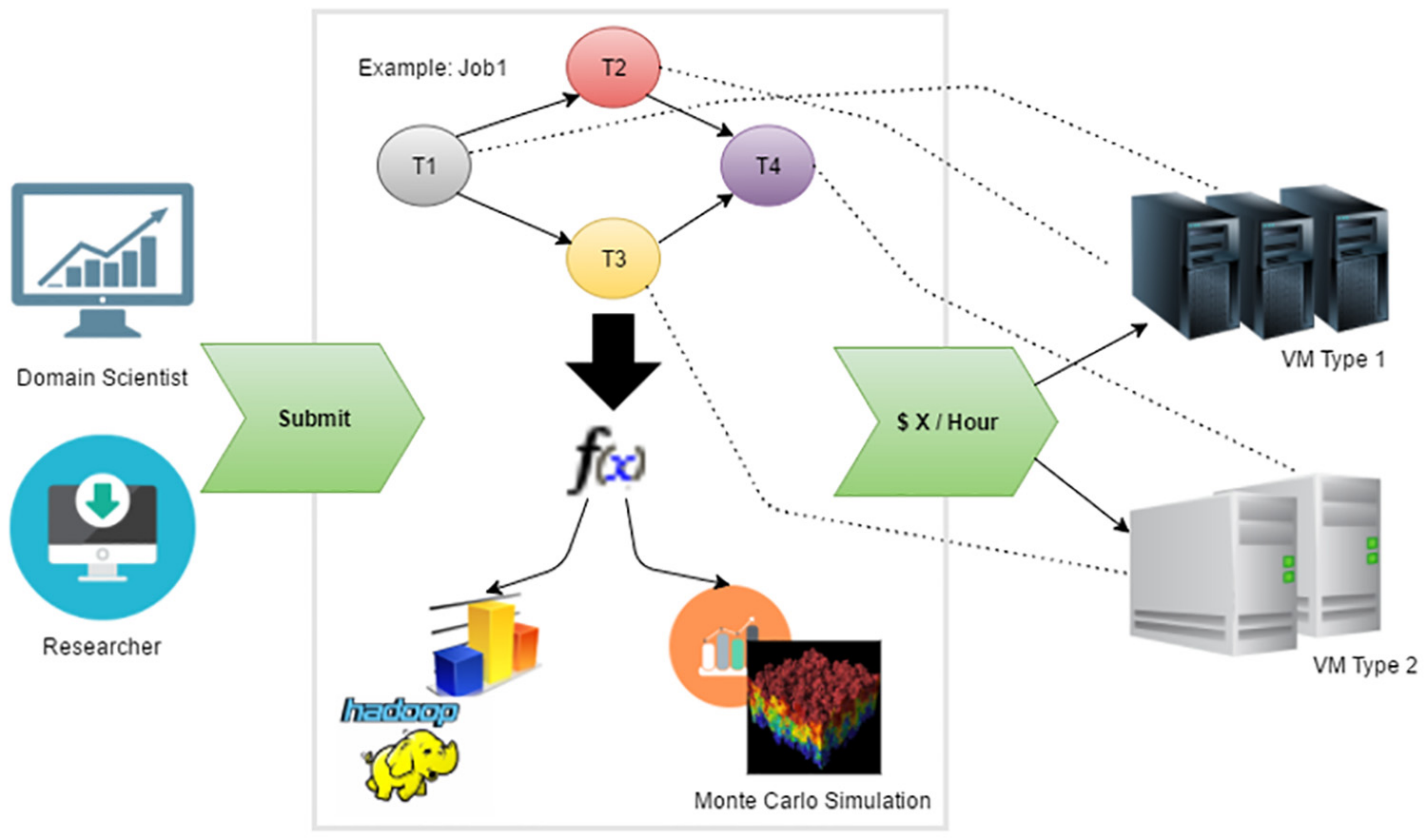
A motivational example.

As a motivating scenario, consider the research wing of the department of environmental science carried a watershed modeling research in the cloud. Firstly, the domain scientists collect and process a large amount of data from copious field observation sites. Then they perform model calibrations to find out the appropriate equation parameters to describe the watershed conditions. They also perform Monte Carlo simulations to predict future watershed movements. As a budget sensitive research center, they decide to move the computing into the cloud and process the research workload using cloud resources. The IT team therefore builds the computing infrastructure in the cloud and runs the application with a budget constraint (e.g. $10/hour). The scientific application is built on an IaaS cloud infrastructure, such as Amazon EC2 and Windows Azure. The VMs in the IaaS architecture allow users to take full control of the software stack, install customized binaries and make persistent configuration changes. These customized OS images can be saved and reused, which ideally serve as the prototypes for the scalable services or components. This application will process workflow jobs submitted by the students, domain scientists and researchers, as well as other collaboration organizations. For this kind of scenarios, they need an auto-scaling mechanism to minimize the job turnaround time and at the same time make sure the running cost does not exceed the budget constraint, i.e. to get the fastest performance for all the jobs within the budget cap. In such kind of cloud application, the assumption is that the workload is combination of workflow jobs that are continuously submitted by different researchers [13]. These tasks in each workflow need to be processed in order and the tasks may have different execution times on different types of VMs. For example, a compute intensive task runs faster on a high-CPU machine than on a high-I/O machine. These machines have different prices. Therefore, a task may prefer one machine type over another considering different metrics, such as the execution time, cost or cost-efficiency. For example, as shown in Fig 1, job1 has four tasks. While taking the shortest execution time possible into consideration, tasks are scheduled on their preferred machine types. The service providers need to dynamically acquire VMs from the cloud providers based on the workload and within the budget constraints while making the scheduling decisions. The role that a budget aware auto-scaling mechanism plays is to help the service providers to determine the number and the type of the acquired resources and allocate those resources to the workflow jobs in a cost-efficient way to reduce the weighted average job turnaround time.

**Fig 2:**
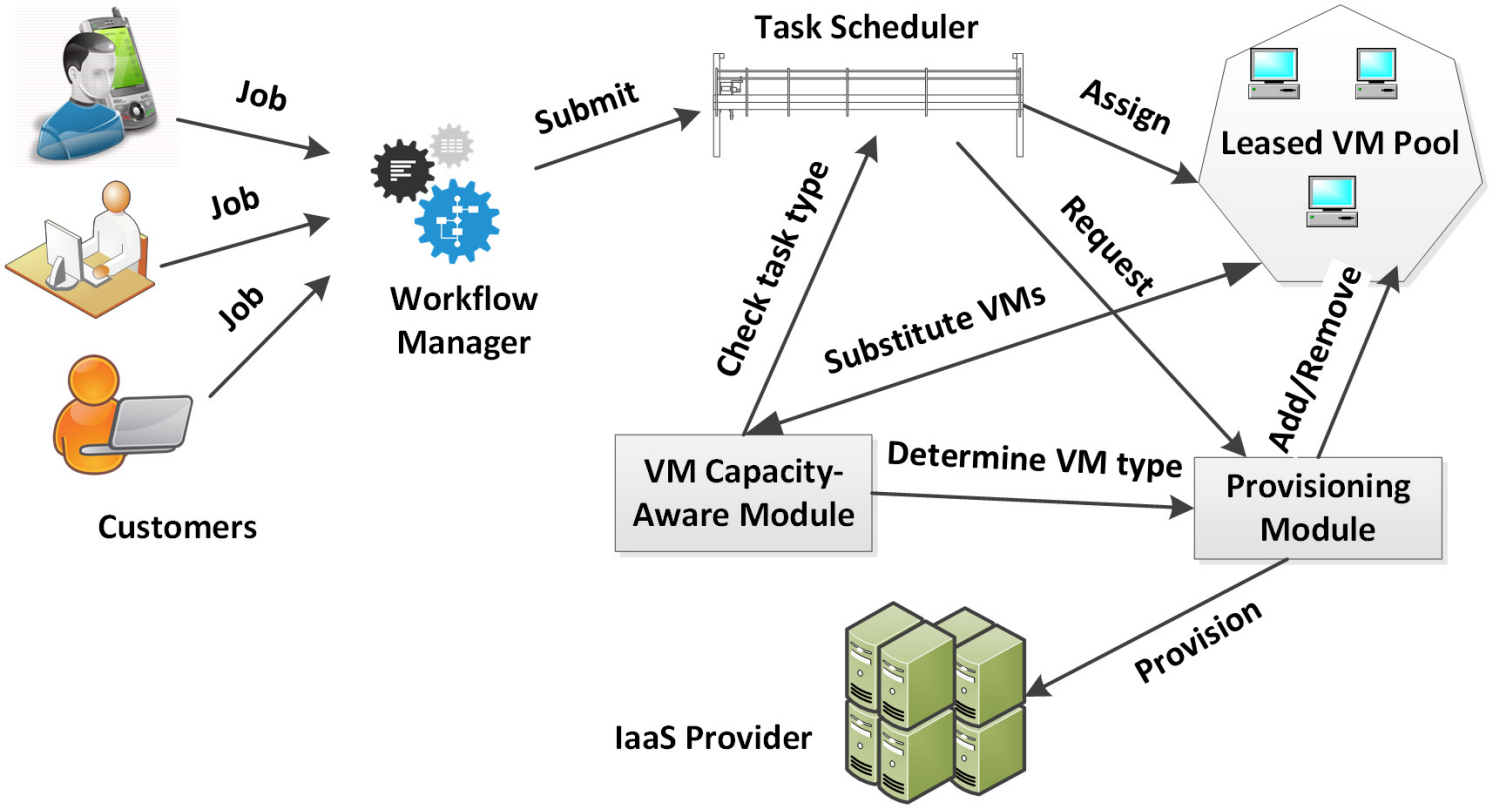
Architectural design of proposed system.

The architectural design of our cloud task scheduler is depicted in Fig 2. Resources are requested and allocated from an underlying IaaS provider. Service customer’s jobs are submitted to the Workflow Manager. User jobs are forwarded to a scheduler that queues component tasks and assigns them to available virtual machines in the pool. System elasticity is enabled by the provisioning module which interfaces with the underlying IaaS provider. This module keeps track of system status information such as queue size or VM status. For each submitted task, it decides whether to launch a new VM or to assign the task to an existing VM.

We assume a resource model similar to Amazon EC2 [11] where VM instances are provisioned on demand. Users are billed by hour, so that, once started, a full hour is charged, even if the instance is partially used. In other words, BTU (Billing Time Unit) is on an hourly basis. There exists heterogeneity in VM capacity with instances, having different CPU power, memory size, and I/O capabilities. A task has exclusive access to an allocated VM instance and there is no preemption until its completion. The proposed framework can target small-scale systems like a university portal service to large-scale scientific computations involving thousands of VM instances.

A job submission invokes the task scheduler, which checks if there are enough resources to accommodate the new arrivals of constituent tasks. If not, it initiates the provisioning of additional virtual machines; the provisioning module calculates the remaining budget and work progress. If more machines should be used, new VMs are launched and the tasks are assigned to them. Another key component is VM capacity-aware module which adds intelligence for provisioning heterogeneous VMs of the underlying cloud infrastructure. It determines the type of VMs to be leased and substitutes existing instances for a better type based on the utilization of each instance type. It is assumed that task execution time on each VM type is somehow known. Tasks may be submitted to the system with some hints regarding their resource requirements, such as CPU, memory, and I/O demands. It is assumed in our work that the task execution time on each VM type is known. For example, task execution time can be determined by using existing performance estimation methods and some heuristic based approaches [14]. The Capacity-Aware (CA) module contacts the VM pool, collecting operational data like the amount of time during which each type of VMs was executing proper tasks and performance deterioration due to the assignment of tasks to non-proper instances.

This paper presents budget-aware and VM capacity-aware provisioning schemes that can be used for cloud applications. Our budget-aware algorithm determines the proper amount of provisioned resources according to the available budget and the progress of the workload. The scheme is further extended into a VM capacity-aware mechanism that provisions different types of virtual machines considering heterogeneity in VM capacity and in task characteristics.

## Budget-Aware Provisioning Scheme

Our Budget-Aware (BA) scheme is an online algorithm that provisions computing resources and schedules tasks at runtime. The idea of BA algorithm is to keep track of the ratio between the remaining budget and work progress and determine proper resource allocations according to this ratio. BA provisioning is based on the budget consumption over the time. A user provides an estimated monetary cost for a job execution prior to the submission. The deadline of a job is defined as the time by which all the constituent tasks of it are to be completed. BA algorithm starts with an amount of resources calculated through the available budget and deadline. Given a budget B in dollars, deadline D in hours, and BTU (Billing Time Unit) price P in dollars, the number of VMs *N*_*vm*_ that can be provisioned not to overspend the budget is determined as follows.

$$\begin{array}{c} N_{vm}=\left(\frac{\left(\frac{B}{P}\right)}{D}\right)*N_{mult} \end{array}$$ (1)

BA provisions up to *N*_*vm*_ machines at the start of the job execution. Then, it periodically computes the ratio between the remaining budget (*B*_*avail*_) and remaining time (*T*_*rem*_) and adjusts the number of the provisioned resources according to this ratio. The higher the ratio, the more resources can be provisioned. The parameter *N*_*mult*_ multiplied with the ratio decides the sensitivity in the resource adjustments, with a higher value implying a bigger increase in the resource lease. Thus, the maximum amount of VMs is determined by Eq (2).

$$\begin{array}{c} N_{vm}=\left(\frac{\left(\frac{B_{avail}}{P}\right)}{T_{rem}}\right)*N_{mult} \end{array}$$ (2)

**Fig 3:**
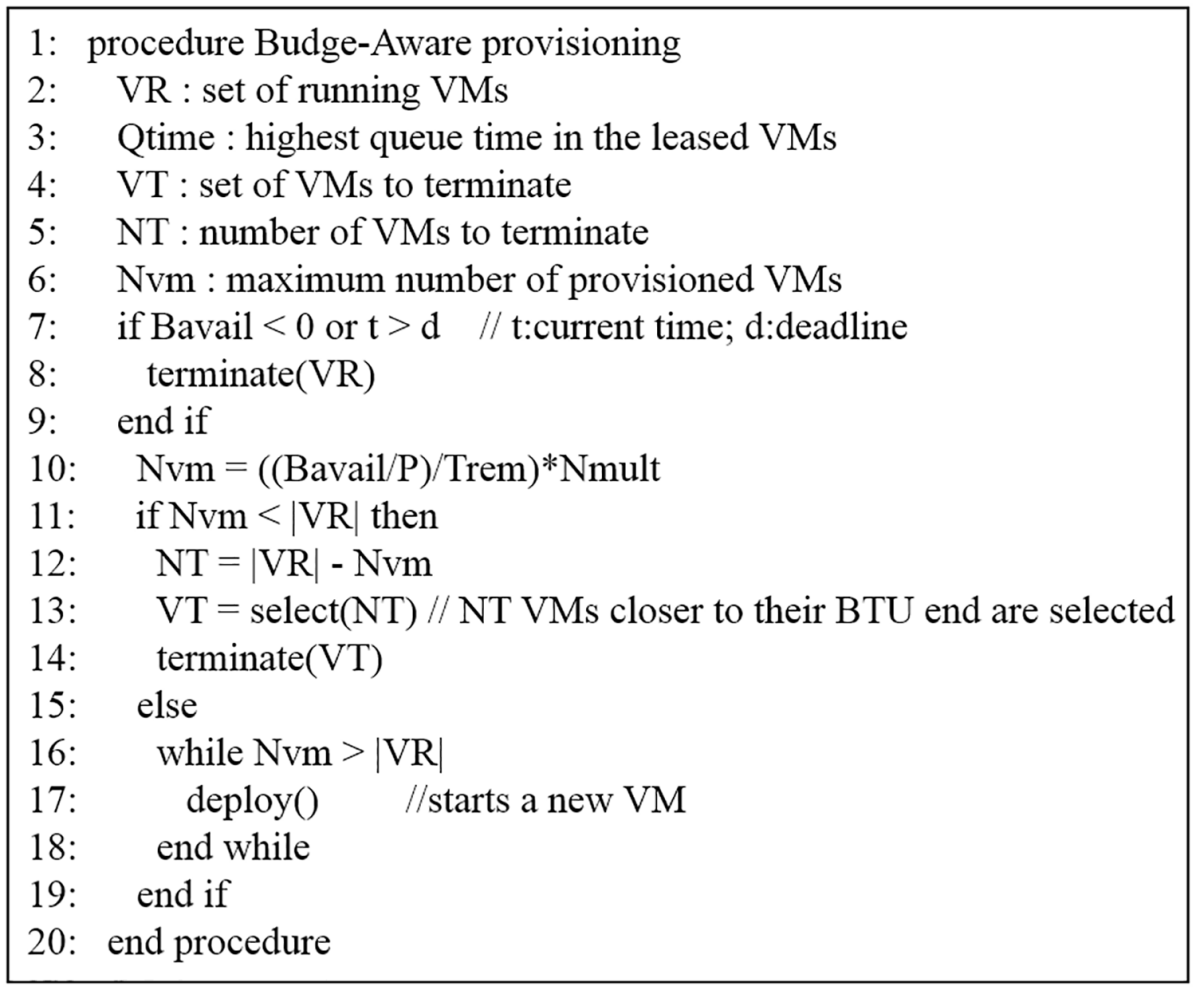
BA provisioning algorithm.

The BA provisioning algorithm is shown in Fig 3. If there is no available budget or the deadline was already passed, all VMs are shut down and the algorithm is terminated (lines 7–9). If the number of leased VMs is less than *N*_*vm*_, more machines can be started to accommodate the tasks that are in the queue (lines 16–18). Otherwise, if the leased resources are more than *N*_*vm*_, some VMs must be terminated. The algorithm chooses VMs that are approaching their hourly billing period, when deciding which VMs to terminate (lines 12–14). If a VM becomes idle, the algorithm terminates it at the end of the current BTU. In an event of VM termination due to an insufficient budget, virtual machines that are closer to the end of their BTU hours are terminated first. However, tasks that are small enough to fit in the remaining time can still be assigned to those machines.

## VM Capacity-Aware Scheduling

BA algorithm is designed for an infrastructure that consists of VMs having the same capabilities. As illustrated in Fig 2, VM capacity-aware module is introduced in order to exploit heterogeneity in task resource requirements and VM instance’s computing power. The CA (Capacity-Aware) provisioning procedure initially distributes the available budget across VM types and adjusts the amount of each virtual machine type according to the nature of tasks contained in the workload. For example, if a larger portion of tasks is compute-intensive tasks, a higher portion of CPU instances are scheduled to perform the computing tasks. The CA scheduling algorithm tries to map the tasks to their optimal machines to minimize the execution time as much as possible, as explained below. We divide the job execution time into two periods: the initial transient period which is from the beginning until the cost of rented VMs reaches the limit (i.e. maximal number of the allowed BTUs), and the stable period which starts after the initial transient period. In the initial transient period, the available budget is divided equally amongst each type of VMs. The budget that can be used per hour should first be calculated. Provided the budget is $55 and the duration of the job is 100 hours, (55/100)*1.5 = $0.82 can be used per hour. Here 1.5 is the value assigned to the parameter *N*_*mult*_ of above equation, which is multiplied with the ratio of budget and deadline to decides the sensitivity in the resource adjustments. Since the budget is distributed equally amongst each type of VMs, the algorithm allocates $0.27 to each instance type. Let us say that a CPU instance costs $0.09, a memory optimized $0.11 and an IO optimized $0.06. Then, 3 CPU instances, 2 memory optimized instances, and 4 IO optimized instances can be leased. The remaining $0.08 is returned back to the system.

In the initial period, there is no information regarding the amount of each type of tasks that comprises the job; thus, CA algorithm equally distributes the available budget amongst each type of VMs. But more accurate instance allocation decisions can be made, as the proportion of each type of tasks contained in the job is being learned of. Characteristics of tasks can be learned through past execution history or using performance estimation techniques [15], [16]. During the stable period, the initial allocation to an instance type is adjusted to be moved to another type. In addition, the system performance is periodically checked to see if any adjustment can lead to a better result. Similar to BA algorithm, the maximum amount of VMs that can be provisioned is determined by Eq (2). The budget that can be used per time unit is first calculated, and new VMs are acquired, until the budget is used up.

**Fig 4:**
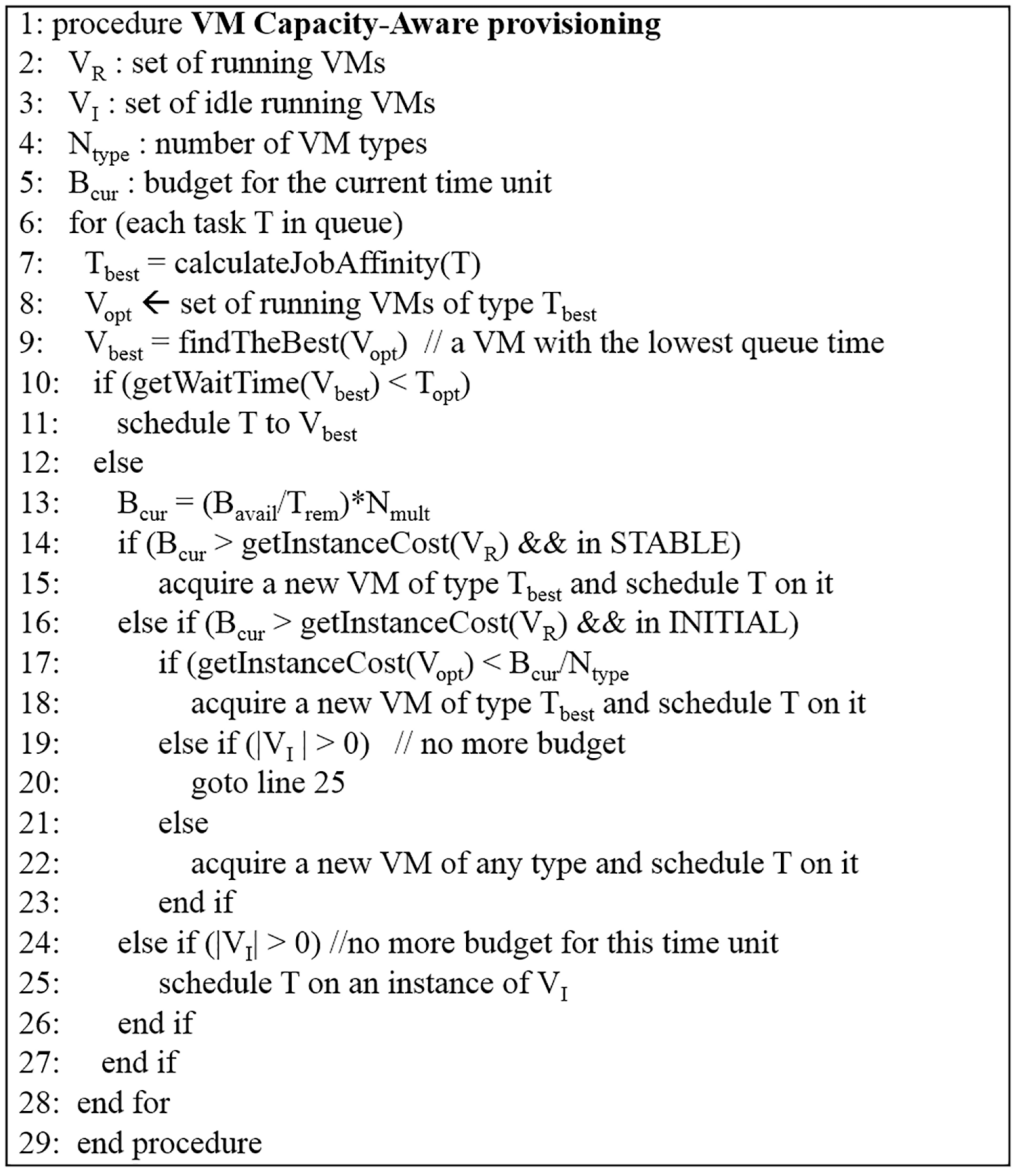
CA provisioning algorithm.

Fig 4 presents our CA provisioning scheme. It is triggered in an iterative way at the event of a task submission or completion. The CA algorithm calculates the job-affinity of next tasks in the queue and schedules them on an instance that has the fastest execution speed (lines 7–11). There may be a case where an optimal VM is not currently available. (In other words, all the instances of the optimal type might be busy running other tasks.) If an optimal instance becomes available in less than *T*_*opt*_, CA algorithm lets the task wait for an optimal instance. The task could be completed earlier than by an instantaneous assignment to a sub-optimal instance. It is noted that job affinity is defined as the relative speed of each instance type for a particular job in this paper. *T*_*opt*_ indicates the maximum amount of time that a task waits in the queue for the instance with the highest job affinity. It is used to prevent assignments of tasks to VMs with lower job affinity, when ideal job-affinity machines will soon become available. In case there is no free optimal instance in less time than *T*_*opt*_, the budget that can be used for the current time unit is calculated and if there is available budget, a new highest job-affinity instance is provisioned (lines 13–15). Otherwise, if the available budget was consumed, the task is scheduled on an idle VM instance regardless of its job-affinity (line 24–26). During the initial period, there is a possibility that an optimal instance cannot be provisioned, as the budget is distributed equally amongst each type of VMs (lines 16–18). In case there are no idle instances to schedule the task, a sub-optimal instance is started (lines 19–23). Instances with the highest job-affinity are referred to as an optimal instance in the algorithm. The job processing speed can be determined during a calibration phase of a job execution or by using performance estimation techniques [15], [16].

After the initial period, the algorithm periodically checks whether a better assignment is possible in terms of machine types, which is not shown in Fig 4. The algorithm defines loss rate as performance degradation of tasks assigned to non-optimal instances. Suppose that a 20 minute task is assigned to a non-optimal VM that needs 30 minutes. Then, the loss rate of this instance type is 10 minutes. *T*_*loss*_ expresses the maximal tolerance level in the loss rate of an instance type. If the loss rate per hour of an instance type increases more than *T*_*loss*_, the instance is switched to the type with the least loss rate. A termination decision of VMs may leave idle time from that point on until the BTU expires. The algorithm substitutes a VM that has idle BTU less than *T*_*sub*_. *T*_*sub*_ expresses a maximum idle time that a VM is allowed to leave prior to termination. It prevents the termination of VMs which have a good amount of available BTU. The two thresholds (*T*_*loss*_, *T*_*sub*_), determine the sensitivity for an instance substitution due to high loss rate.

**Fig 5:**
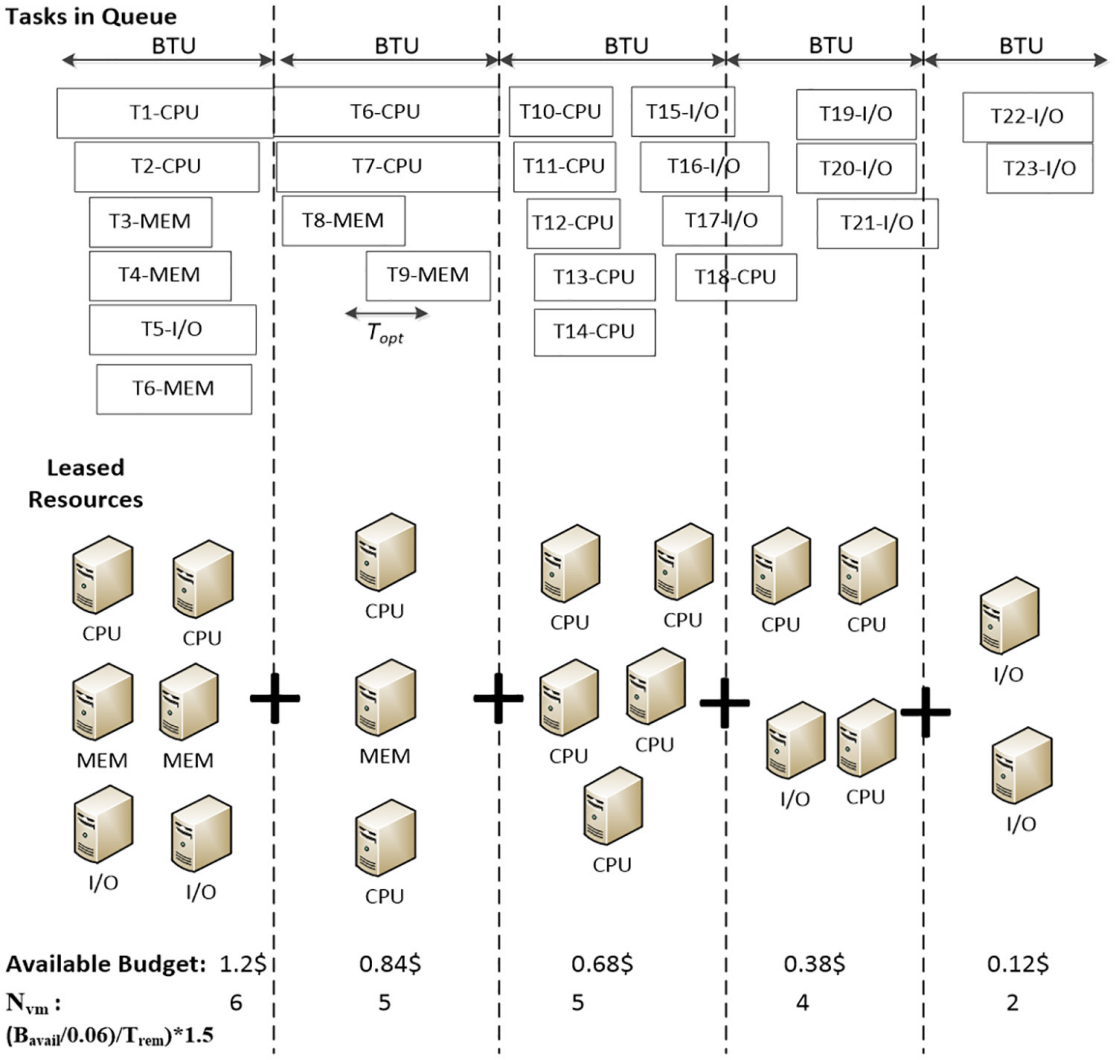
Sample run of VM capacity-aware provisioning.

A sample run of the algorithm is presented in Fig 5. With different types of tasks and various VMs, the budget is distributed amongst each type of VMs. At the beginning, an equal number of VMs of each type, each costing $0.06 per hour, is leased. Tasks T1-T5 are assigned to their highest job affinity VM, and T6 is assigned to an I/O instance, since no additional memory instances can be leased. Because the limit in each type of VM is reached, CA algorithm enters the stable period. In the second time unit, T6, T7, and T8 can be assigned to their optimal instance. T9 is also assigned to the already leased fastest machine, because the queue time for it is smaller than *T*_*opt*_. In the 3rd time unit, 5 CPU instances are launched to accommodate 5 CPU tasks in the queue. Some moments later, 3 I/O tasks are submitted. Since the limit in the amount of VMs is reached, I/O instances cannot be launched. Therefore, the I/O tasks are assigned to CPU instances, increasing significantly the loss rate of the CPU instances. A CPU instance is then replaced with an I/O instance. Additionally, one more CPU instance is terminated, because the limit was exceeded. Similarly in the last period, a CPU instance is substituted and a CPU instance is terminated.

## Performance Evaluation

In order to evaluate the performance of our proposed algorithms, we implemented a cloud workflow simulator based on the CloudSim toolkit [12]. Our simulator consists of Datacenter Broker, Cloudlet, and VM entities. The central piece of it is the Datacenter Broker that models provisioning policies and algorithms. The Cloudlet entity represents a task submitted to the cloud. The entity is extended by adding a label which describes the type of the task (i.e., hint). The Datacenter Broker manages the provisioning of VMs and the scheduling of tasks. On receipt of tasks in the form of Cloudlet entities, it makes decisions to acquire or terminate VMs. The broker also monitors the runtime workload information and decides to which VM to assign a task based on a pre-specified policy. A delay in the execution time of a task is inserted, if it is assigned to a VM of non-optimal type, which simulates the performance deterioration of a task assigned to an improper type of VMs. The VM entity includes information such as queue time, available time before the end of BTU, and a type label. It is assumed that VMs have a single core and execute sequentially the tasks. We have added a mechanism which generates synthetic workloads with different types of tasks. The Datacenter Broker reads the list of the submitted tasks and allocates the tasks to VMs. Each assigned task is removed from the submitted list and the Datacenter Broker runs in an iterative way, until all the tasks are submitted and executed.

### Simulation Workloads

We evaluated the proposed algorithms against two representative workload patterns: bursty and steady. The bursty pattern features spikes of intense activity with periods of mild activity between the peaks, while in the steady pattern jobs arrive in a relatively steady and consistent manner. The bursty pattern can represent a scenario where a news or video becomes popular and fades away quickly. The steady pattern represents an application that tends to run all the time in a steady state. We consider synthetic, bag-of-tasks workloads with runtimes typical for data mining and semi-interactive processing [17]. Two types of steady workloads are used, one with many short tasks and the other with longer tasks. The first workload consists of tasks with the runtime of 1-8 minutes and the submission rate of 60 tasks per hour. The second steady workload consists of longer tasks with the runtime spread over 5-25 minutes. 20 tasks are submitted each hour. Similarly to the steady workloads, we used two bursty workloads: one with short tasks and the other with longer tasks. The bursty workload alternates 10 periods of 180 minute steady arrivals and 60 minute busty arrivals. Workloads are composed of CPU-, memory-, and I/O-intensive tasks. Task runtime degradation from a non-optimal assignment is 25%, which is considered as reasonable performance deterioration for a task being executed on an inappropriate VM [18].

### Alternative Provisioning Schemes

Our budget-optimized provisioning strategies are compared with notable resource provisioning policies [19], [20], [21]. Most existing resource provisioning approaches aims to reduce the cost of job executions, likely decreasing job response time without considering how much users are willing to spend for their job. Thus, the current schemes are unsuitable for scenarios where a pre-defined budget is one of the key factors. Each of the schemes is briefly summarized below.

- Steady Stream (SS) scheme launches new VMs, when the total queue time of the jobs becomes five times greater than the estimated waste time. Otherwise, it assigns an incoming job to a VM with the least queue time. The waste time is the sum of the times required to boot and terminate a VM. The algorithm is adjusted to be driven by an on-demand pricing model (, which terminates VMs only when they finish their BTU.) In addition, the algorithm is modified to function in environments with heterogeneous VMs; tasks are assigned to a VM with the least queue time, if the total queue time does not exceed certain times the waste time. Otherwise, it starts an optimal machine.
- Relaxed (R) algorithm controls a trade-off between cost and delay by adjusting the bound on the queue time. If the queue time of an arriving job does not exceed a few times the job runtime, the job will be mapped to an already running VM. Otherwise, a new VM will be started to execute the job. Similarly to SS case, a configuration with a higher bound in the task queue time will decrease the total cost for a higher slowdown.
- Scaling-First (SF) algorithm aims at a heterogeneous environment. Its behavior is similar, in spirit, to our CA. But it calculates its maximum budget that can be used within the next time unit and always acquires the same amount of each resource type.

A primary goal of our simulation study is to assess the performance of our proposals. For that purpose, our algorithms are simulated under different settings such as scenarios with tight budgets, bursty workloads, or heterogeneous infrastructures. As a key performance metric, we define slowdown as the time a task waits in the queue together with increased runtime due to non-optimal task assignments. Note that the runtime increase is a difference between the execution time of a task on a non-optimal instance and an estimated execution time by an optimal instance. Remaining budget metric indicates whether the provisioning algorithm can execute the workload without surpassing its budget. Through a set of simulation cases, it has been proved that our proposals are cost-beneficial and capable of running tasks within a defined budget in both homogeneous and heterogeneous environments.

### Performance of BA Provisioning Scheme

We first look at the performance of our budget-aware algorithm and compare it with that of SS and R schemes for an infrastructure composed of homogeneous VMs. In such environments, all VMs are equipped with identical capability; all VMs have the same CPU, memory, and I/O capability. The performance results are summarized in Table 1. It demonstrates that our BA scheme can execute tasks within a budget, with similar cost and performance to other competing algorithms. With either steady or bursty workloads, our BA algorithm manages to complete all the tasks within the budget limit; tasks are handled using less BTUs than others, but with comparable slowdown rates. A configuration with a smaller initial budget for BA case or a tighter bound in the job queue time for SS case can decrease the execution cost. In either case, a higher slowdown rate is incurred because the algorithms tend to assign tasks to already acquired machines rather than to provision new ones. Besides, R algorithm cannot be configured to run on settings that incurs a low execution cost, as the amount of the provisioning resources depends on job runtimes rather than on controllable parameters such as the budget or the bound in the task queue time. Therefore, it is important to know that a direct comparison among the candidate schemes may provide a limited insight into their performance. It is because BA algorithm dynamically adjusts resource provisioning based on a desired budget, while SS and R schemes consider parameters such as queue time or task runtime, when making their provisioning decisions.

**Table 1:** Performance comparison of provisioning schemes.

BA				SS			R		
*Budget*($)	Remaining($)	Cost(BTU)	Sd(min)	QT(min)	Cost(BTU)	Sd(min)	QT(min)	Cost(BTU)	Sd(min)
13	3.15	165	0.20	1	189	0.07	1	170	4.51
12	0.32	166	0.32	5	176	1.48	2	173	7.95
11	1.15	164	0.54	10	173	4.21	3	167	11.80
10	0.22	163	0.56	30	168	14.15	0.7	169	2.81
9	0.06	149	19.13	40	164	16.91	0.5	172	1.93

Table notes: Sd represents Slowdown, and QT stands for Queue Time.

In contrast, our CA algorithm, which is an extension of BA provisioning scheme for heterogeneous VM compositions, can directly be compared to SF and a modified version of SS scheme. SS algorithm can be easily adapted to run on a wide range of execution costs by adjusting the bound in the task queue time. Since R’s provisioning decisions depend on job runtime, the algorithm cannot be configured to run a workload on a particular range of execution costs. Therefore, the performance of our CA algorithm is compared only with that of SS and SF schemes in the following subsection.

### Provisioning Performance for Heterogeneous VMs

The potential of CA algorithm can be mostly demonstrated on an infrastructure with different types of resources. The simulation setup consists of 3 VM types: compute-optimized, memory-optimized, and I/O-optimized VMs. The performance of CA algorithm is compared with that of SF and SS schemes. To evaluate different compositions of task heterogeneity in workloads, we repeated our simulation runs in three settings of different proportions of task types. In the first scenario, 70% of the tasks are CPU-intensive, and the remaining 30% is equally split between memory-intensive and I/O-intensive tasks. In the second scenario, CPU-intensive tasks comprise 50% of the tasks in the workload and the remaining 50% is also evenly divided between memory-intensive and I/O-intensive tasks. The workload for the last scenario consists of an equal amount of 33% for each task type.

**Fig 6:**
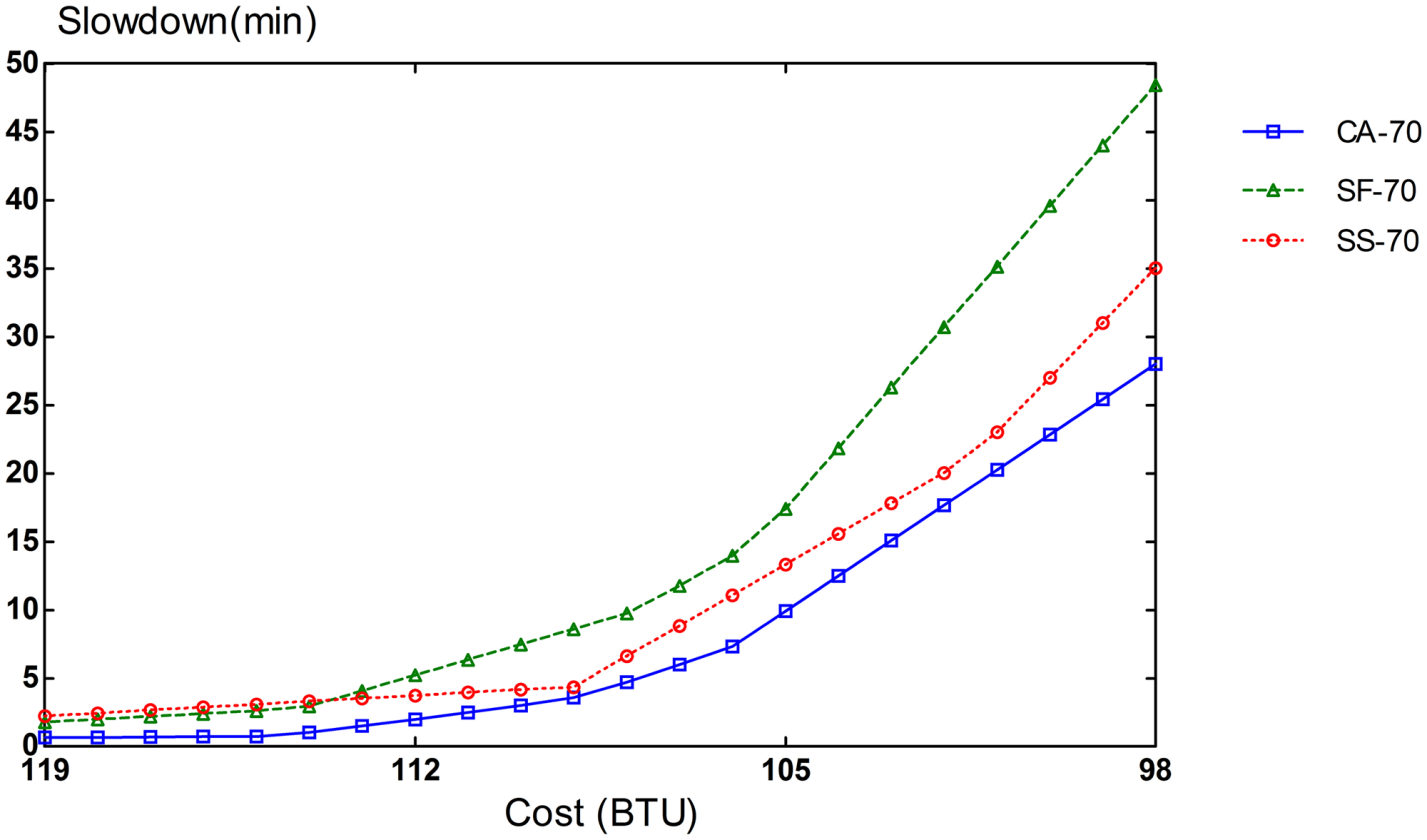
70% of CPU tasks for steady workload.

**Fig 7:**
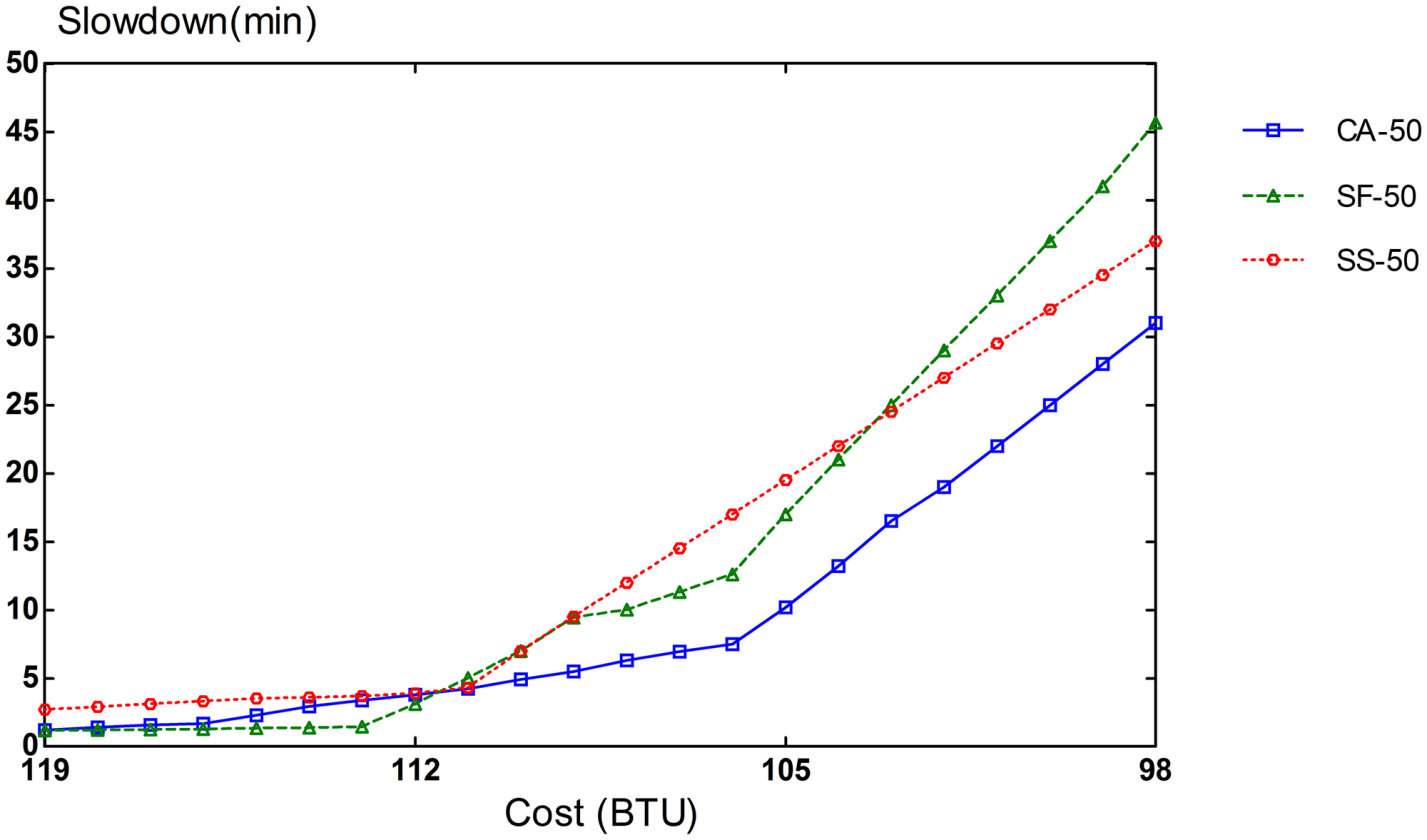
50% of CPU tasks for steady workload.

**Fig 8:**
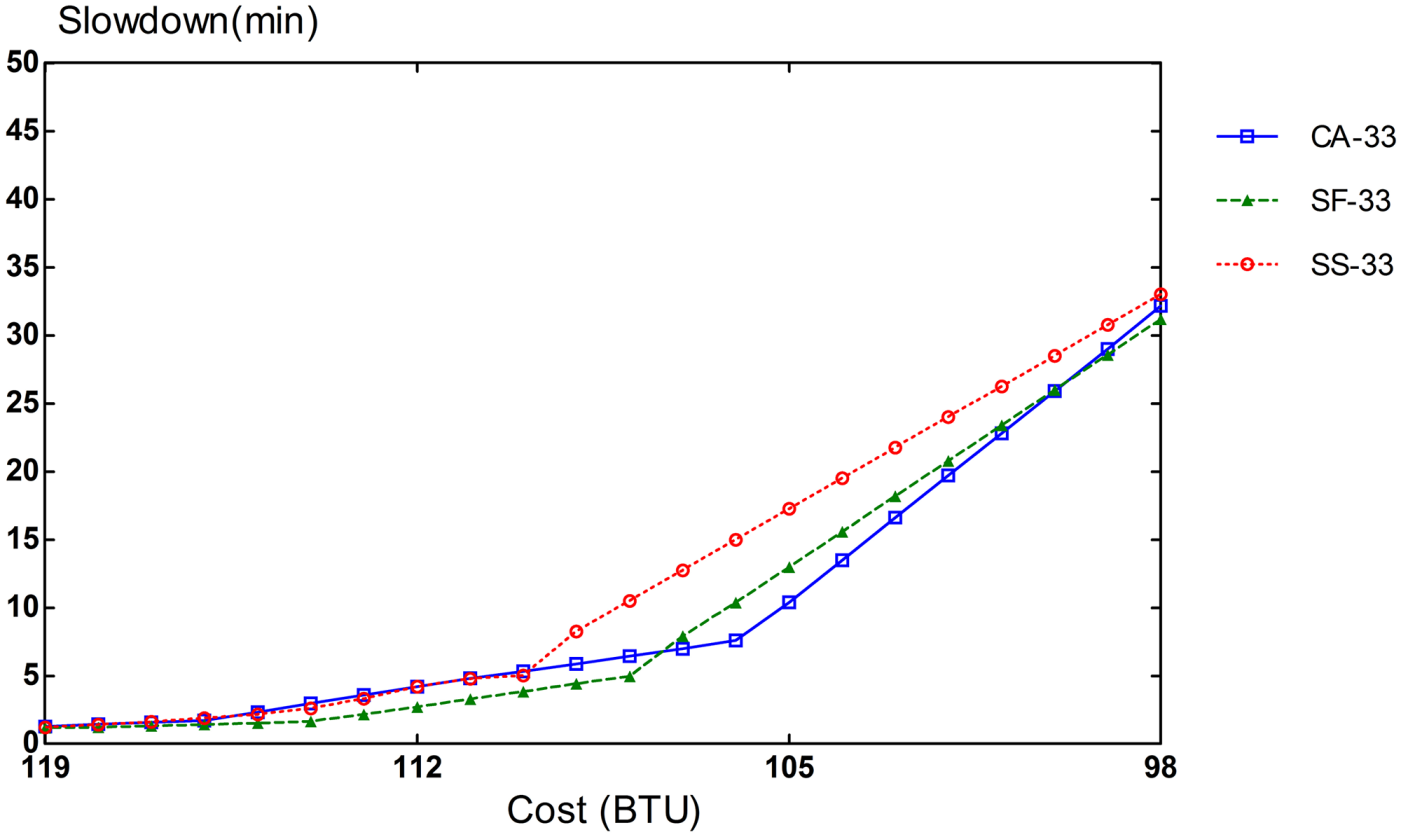
33% of CPU tasks for steady workload.

**Fig 9:**
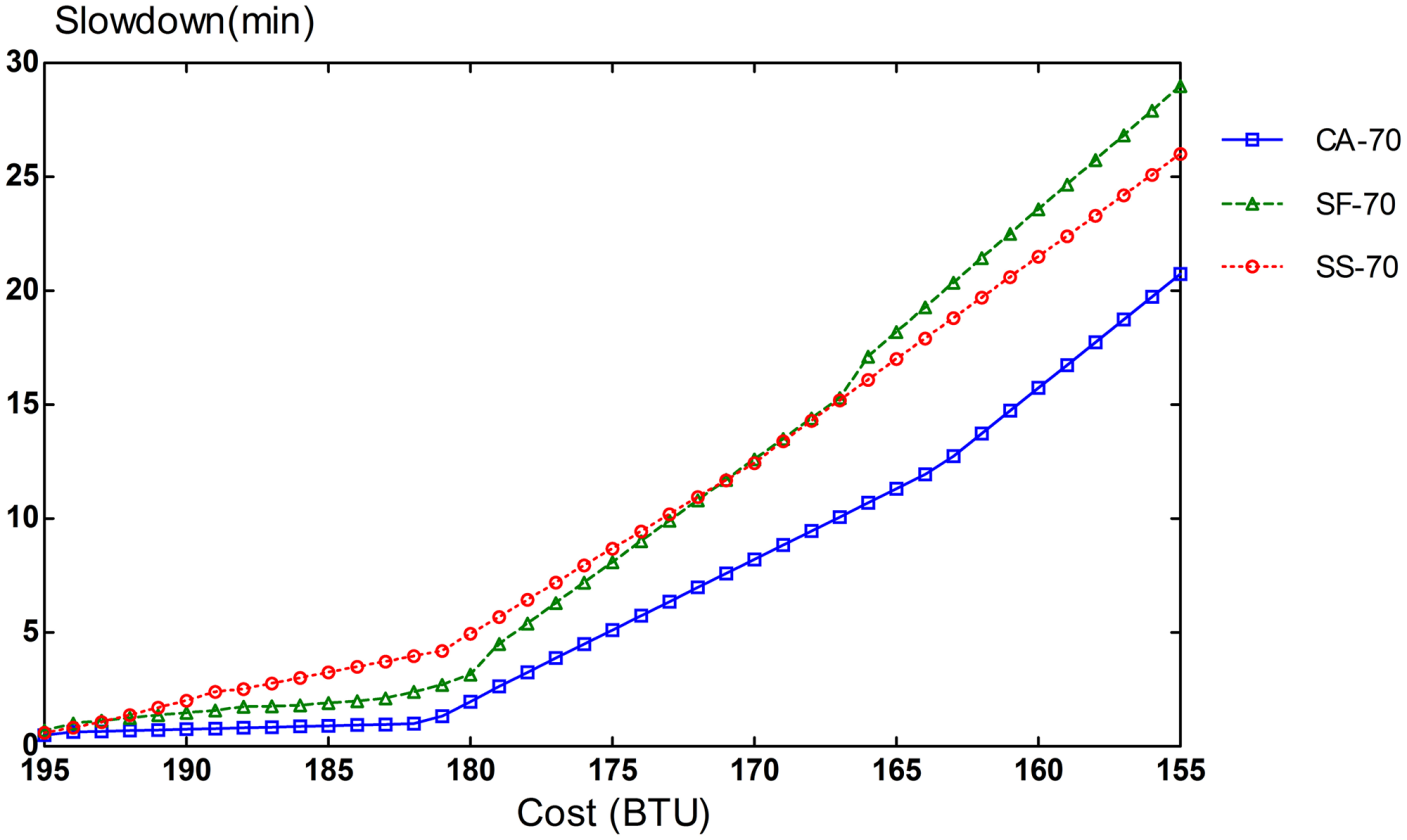
70% of CPU tasks for bursty workload.

**Fig 10:**
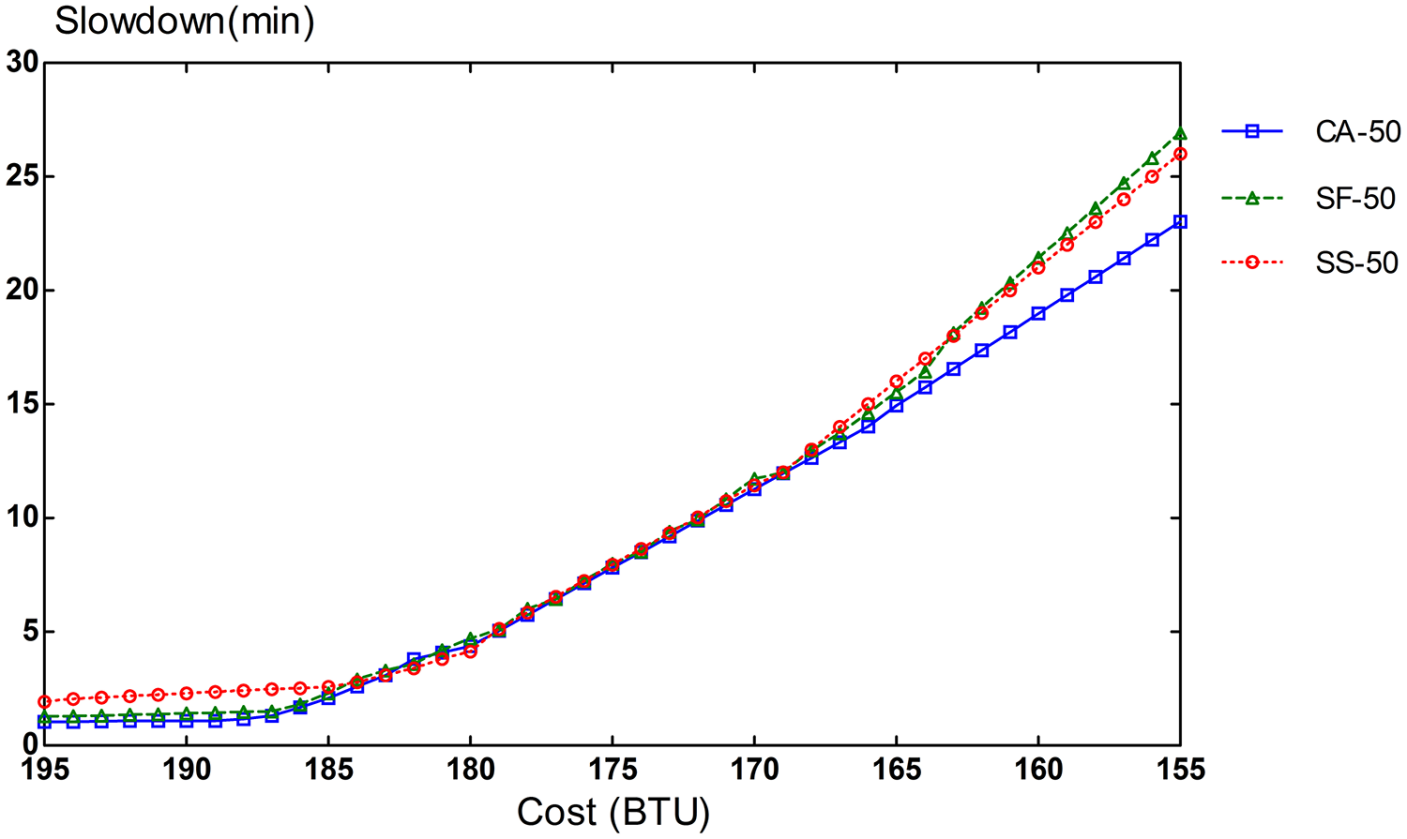
50% of CPU tasks for bursty workload.

**Fig 11:**
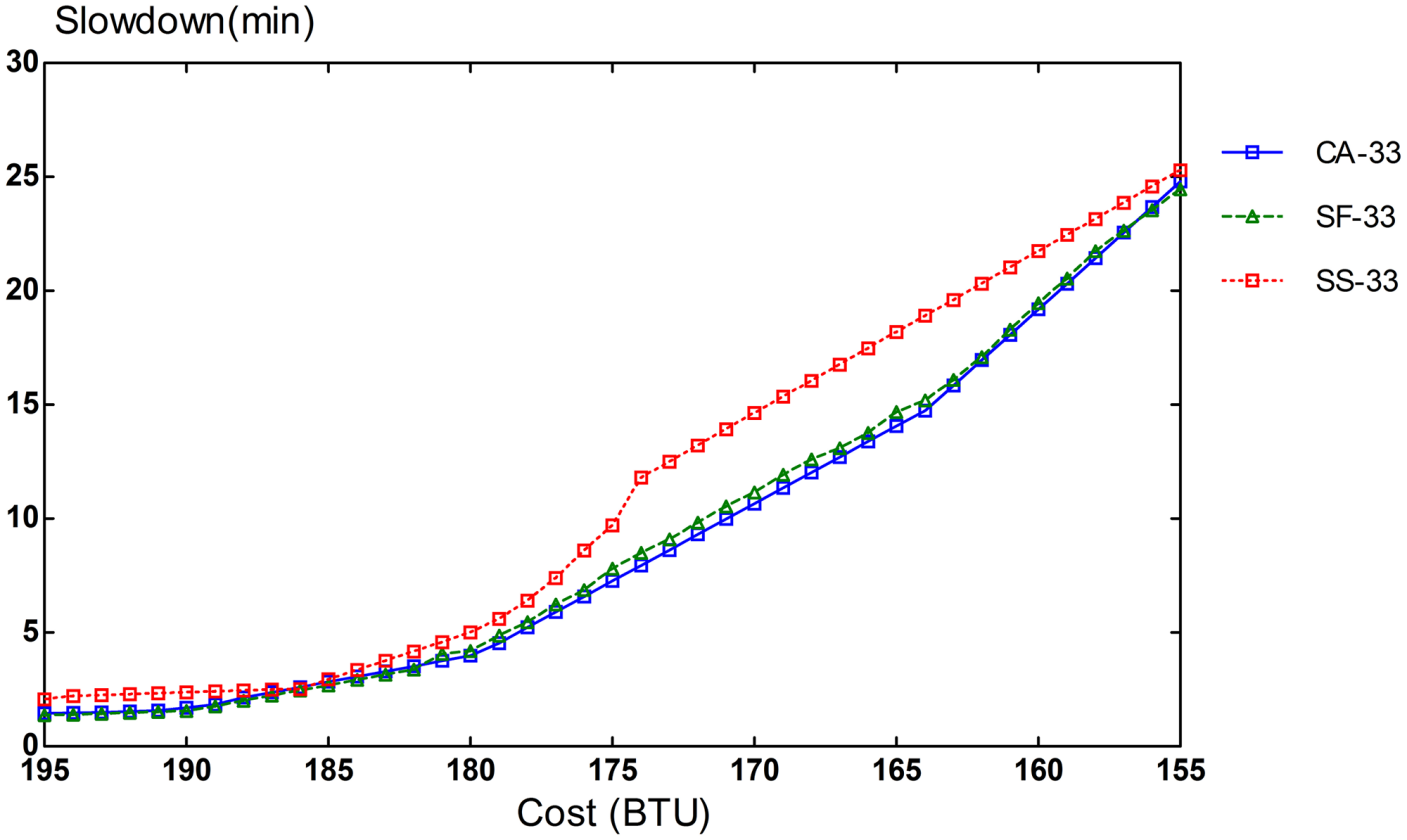
33% of CPU tasks for bursty workload.

Slowdown rates of provisioning schemes for steady and bursty workloads are presented in Figs 6, 7, 8, 9, 10 and 11. The graphs represent the different percentages of CPU-intensive tasks for CA, SF, and SS algorithms. For example, CA-70 refers to CA algorithm running in the first scenario, while SS-33 refers to SS scheme with 33% of compute-intensive tasks. It has been observed that CA algorithm consistently outperforms SF and SS schemes for both scenarios, whether the budget is flexible or tight. As expected, there is a performance gap between CA algorithm and others in the case of 70% of compute tasks. This is because it allows for CA scheme to take advantage of VM heterogeneity when adjusting its resource allocations across different types.

Our CA algorithm reveals a significant gain in the performance compared to SF and SS cases with a smaller budget. A bigger budget allows a higher amount of resources to be allocated; SF scheme is also able to select optimal type resources. Similarly, SS algorithm is given enough chances to assign tasks to optimal VMs, when configured to use more resources to execute the workload by decreasing the bound in the queue time. In contrast, when the budget is tight, the number of resources that can be provisioned is lower, and the selection of optimal resources will likely have a larger impact on the slowdown rate. When the cost nears the smallest budget required for the workload, CA scheme achieves 20-70% gain in the performance depending on the percentage of task heterogeneity compared to SF case. A lesser gain of 15-30% is expected against SS algorithm. It is noted that the performance gain grows, when the percentage of input task heterogeneity becomes higher.

In addition, CA algorithm achieves a higher gain from SF scheme for steady workloads. The reason behind this is that, given bursty workloads, the slowdown caused by the waiting time of tasks in the queue increases significantly during bursts of task arrivals. In other words, the slowdown incurred by the waiting time of tasks in the queue due to resource unavailability is noticeable, adversely affecting the overall slowdown rate of both CA and SF schemes. In the case of steady workloads, the delay in the queue is not that important; SF’s performance gain is mainly attributed to the assignment of tasks to proper instances. Therefore, with steady workloads, the performance gain of CA algorithm is higher, because assignments of tasks to non-optimal instances have a bigger impact in the overall slowdown of SF.

**Fig 12:**
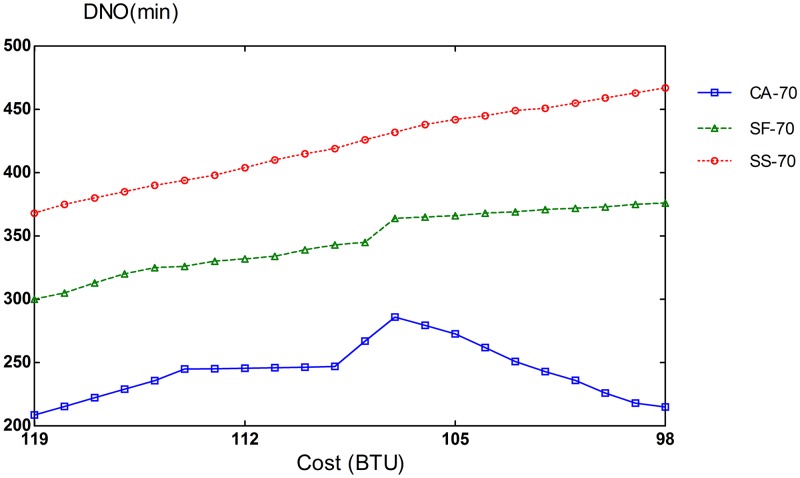
Performance degradation for 70% CPU instances.

**Fig 13:**
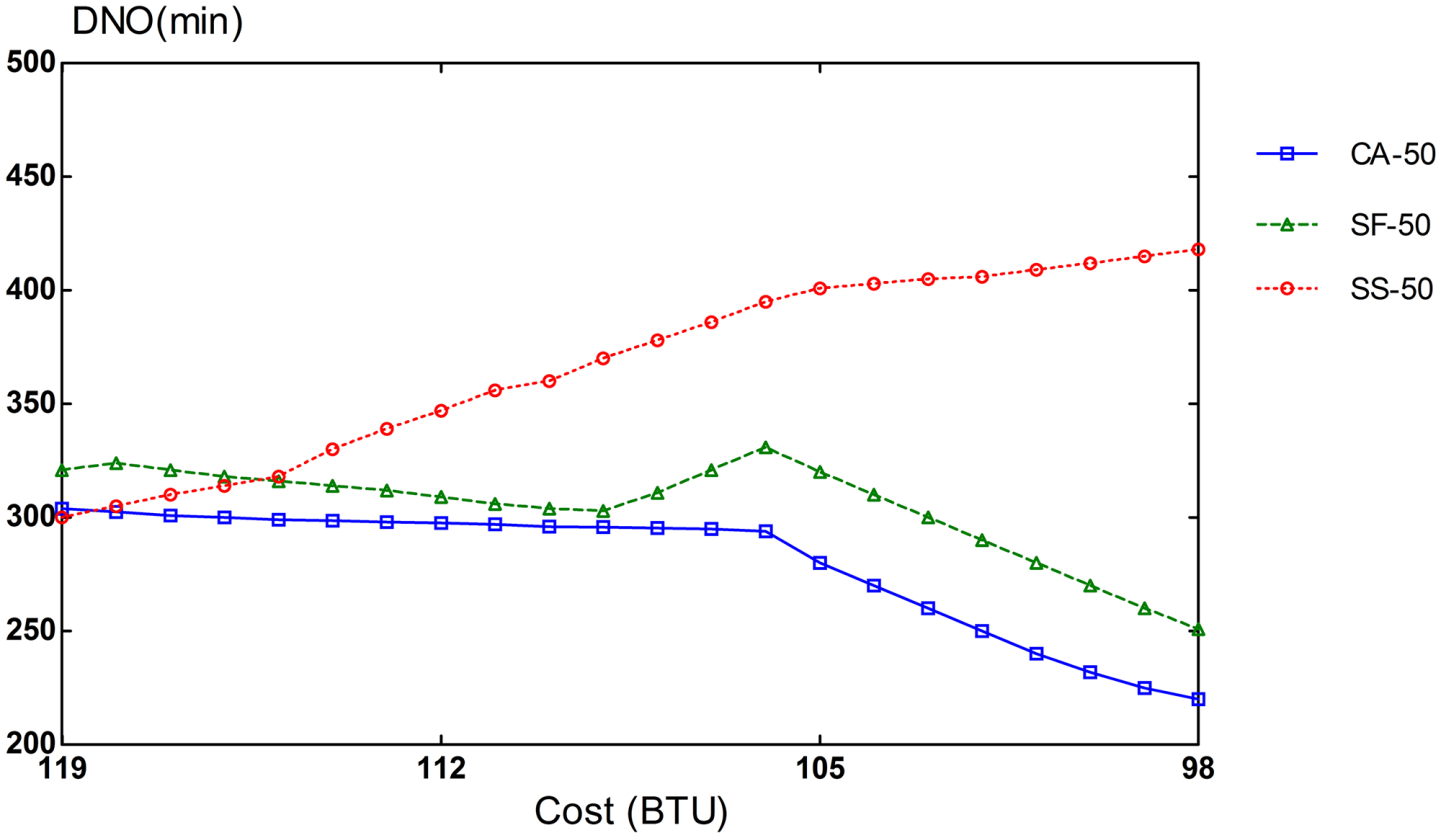
Performance degradation for 50% CPU instances.

**Fig 14:**
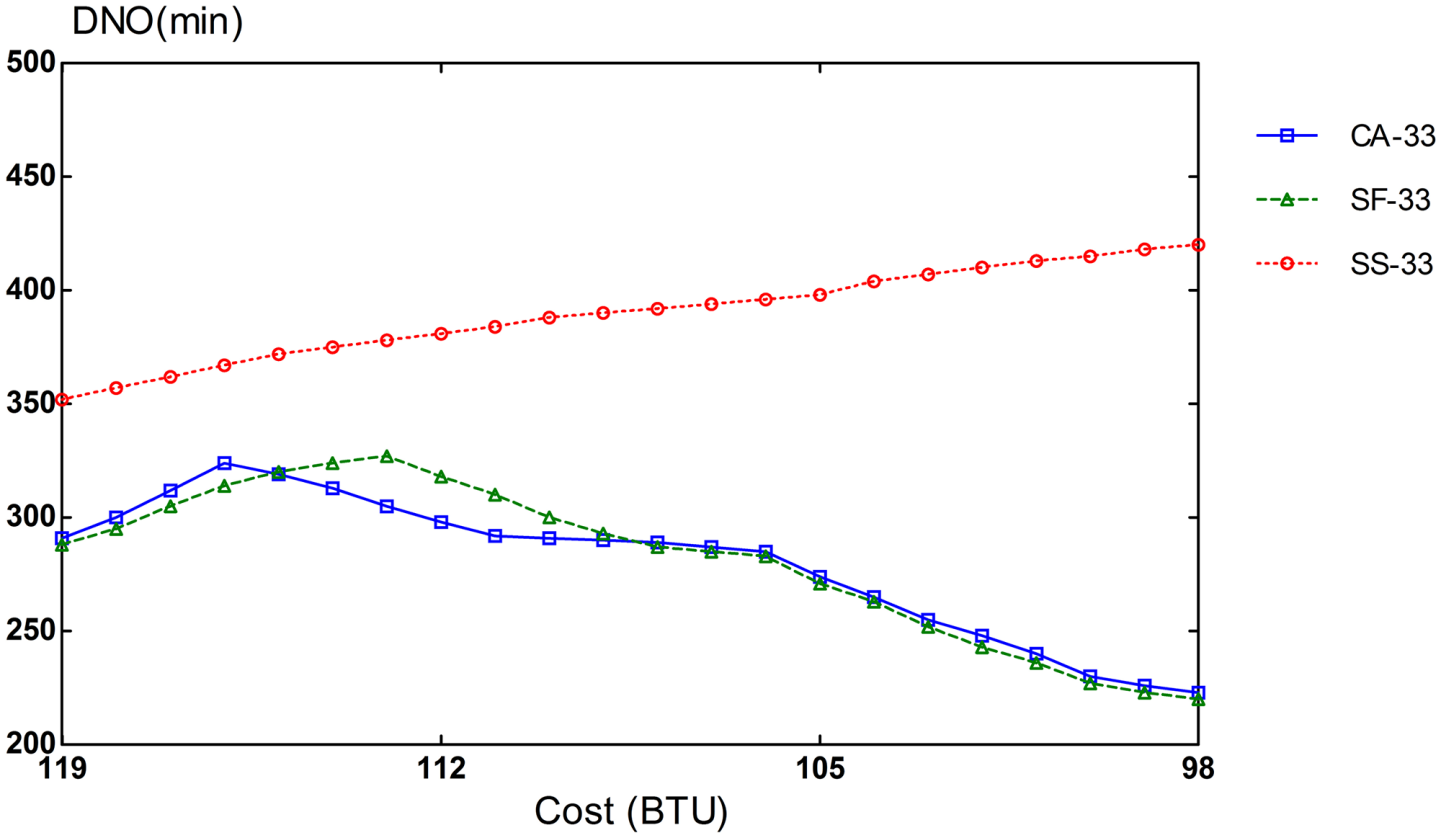
Performance degradation for 33% CPU instances.

There are two important factors that can cause performance degradation. One is the waiting time in the queue due to resource unavailability, and the second is the assignment of tasks to sub-optimal instances. We measure the degree of performance degradation caused by non-optimal task assignments. The deterioration degree is gauged in the metric of DNO (Degree of Non-Optimal tasks assignments). DNO is defined as an increase in task runtime due to sub-optimal VM assignments. For instance, if a 5 minute compute-intensive task is assigned to a memory-optimized instance that will take 7 minutes to execute the task, the DNO becomes 2 minutes. As shown in Figs 12, 13 and 14, our CA scheme reduces DNO significantly by considering the type of tasks in workloads, when provisioning resources. CA algorithm reduces performance degradation by non-optimal assignments on average by 27% and 60% compared to SF and SS, respectively. These results are for the case of steady workloads. It is also noted that bursty workload cases exhibit similar patterns. Similarly to the slowdown rate, a greater heterogeneity in VM types leads to a larger performance difference between CA and SF cases. The results once again confirm the importance of provisioning and scheduling appropriate instance types for input tasks in the workload.

**Fig 15:**
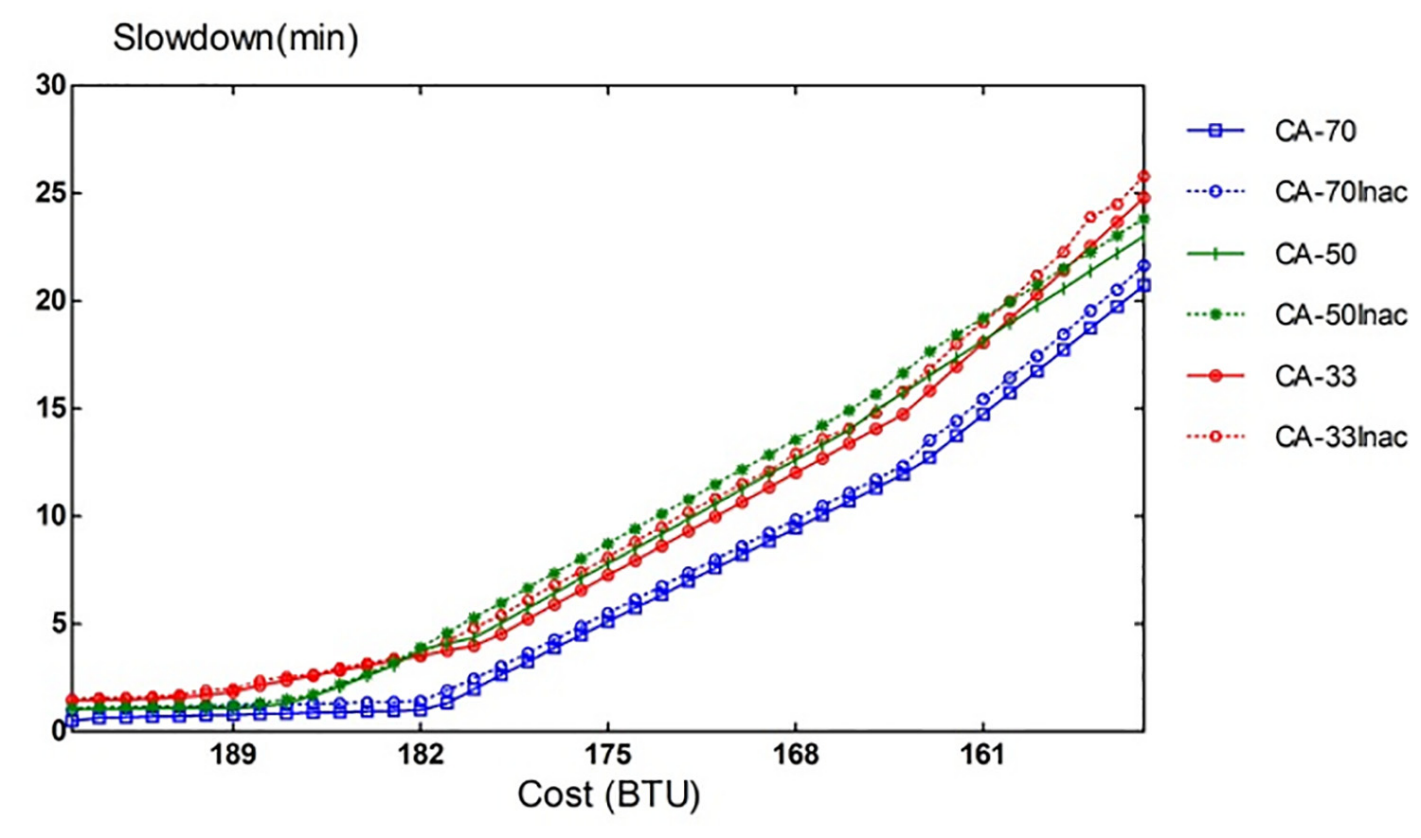
Sensitivity to runtime estimation accuracy.

Our CA algorithm assumes that task runtime can be estimated prior to its execution. In practice, however, such information could not be always accessible or accurate. Therefore, this part of the simulation looks at impact that inaccurate task runtime estimation can have on the performance of CA algorithm. Fig 15 shows the performance, when errors of ± 20% range are intentionally inserted. CA-70-inaccurate case shows the slowdown rate of CA scheme in the presence of the estimation errors. We can see that the performance degradation from inaccurate runtime estimations is contained to a certain extent. This is because CA algorithm uses task runtime information only when deciding whether to assign the task to a VM with enough remaining BTU. Additionally, task scheduling is performed dynamically at the event of a task assignment or completion.

## Related Work

There have been a number of recent efforts focusing on the use of cloud computing for data-intensive applications [22], [23]. Similarly to ours, some of them tackles the problem of resource provisioning to execute workflow tasks in a single IaaS cloud [19]–[21], while others present scheduling strategies for cloud-augmented clusters or hybrid cloud environments [24]–[25]. One of the most relevant to our provisioning scheme can be found at the resource manager that dynamically leverages cloud resources in response to different task submission patterns [19]. The study investigated several strategies to allocate a virtual machine from the cloud, when there are no available VMs. Unlike ours, their priority is to minimize execution delay together with the number of idle VMs and waste time caused by the constant launch and termination of VMs. Also, their workloads were dominated by short running tasks, since the waste time is small, unlikely making any noticeable difference for scheduling long running tasks.

A commercial cloud provisioning of on-demand resources, homogeneous network, and pay-as-you-use pricing model using prediction of the completion and transmission times to minimize the workflow execution cost while meeting the deadline-constraints of the workflow is presented in [26]. And a set of strategies based on classic heuristics for online scheduling and bin-packing problem are investigated [20]. An on-demand pricing model is proposed, in which a trade-off between cost and performance is explored by delaying job executions for VMs that would become available some time later. The partial critical path (PCP) algorithm [27] tried to minimize the cost while fulfilling the user defined deadline of a given workflow. The algorithm works in two phases: in first phase, it assigns subdeadlines to the critical path tasks; while in the second phase, it schedules tasks in the economic available resources that can gratify the subdeadline constraints. In [28], a service-oriented workflow scheduling algorithm that smears hybrid metric based on recommendation trust and uncertainty of workflow scheduling in cloud. The underlying technique of the system is to fine-tune the weight of cost incrementally until the execution time of all the tasks satisfies the deadline. The study concentrates on making a satisfactory trade-off between speed and cost, while our CA algorithm focuses on the execution of a workload within a defined cost. In their approach, it is difficult to set the target monetary cost to complete a workflow job, as the bound on the queue time to launch a new VM instance determines the actual cost. Cloud customers may not have adequate knowledge to interrelate the monetary cost and the bound placed on the queue wait time. A performance-cost analysis for a set of provisioning and allocation strategies is also relevant to our work [29]. Separate queues for each range of job durations are introduced, so that higher priorities are given to the queues for shorter jobs, which reduce the slowdown of shorter jobs. However, it may cause significant delay or even starvation for long tasks, although the overall slowdown was able to be reduced by favoring shorter tasks. In addition, a cost model considered in their work is proportional to the runtime of VM instances. An analytical cost-performance framework based algorithm for service deployment in hybrid clouds which focus on the dynamic optimization of public and private nodes provisioning across the services while taking performance-cost tradeoff in to consideration was investigated in [29]. We consider an on-demand cost model that leaves a VM charged for one hour after its acquisition, even if there is no further work.

Additionally, an optimal provisioning scheme for a priori known workflow ensembles with varying deadline and budget constraints is considered [30]. Their dynamic provisioning algorithm starts a fixed number of resources based on available time and budget. Then, the resource allocation is adjusted accordingly to how efficiently they are utilized. In contrast to our CA algorithm, it tries to maximize user-prioritized workflows that can be completed within a given budget. In other words, some jobs can be preferably scheduled ahead of others. A priority in the work is placed not on executing all workflow tasks, but on completing the tasks that have higher precedence. Budget-constrained Heterogeneous Earliest Finish Time (BHEFT) was proposed in [31] as an extension of HEFT algorithm [32]. BHEFT delineate an appropriate blueprint by reducing the make-span so that the user’s budget and deadline constraints are fulfilled while considering the load on each provider in the presence of multiple and heterogeneous service providers. They used success rate as metric to find out the percentage of problems for which the plan was found as in opposing case of the failure in satisfying both the constraints (i.e. budget and deadline). Two task allocation algorithms were proposed to minimize the delay within budget constraints [21]. One of them is the scaling-first algorithm that calculates the cost of resources needed within the next hour and acquires resources proportionally for each VM type. Then, it schedules jobs on the fastest machines based on their priority. Although it is argued that their approach targets an environment with different types of VMs, the instances could differ only in their computation power. In other words, their scheme does not consider differences in other aspects of the resources such as high-speed memory, networks, or GPUs. Additionally, a fixed amount of budget is assumed in their work for every time unit during the entire span of application executions.

Scheduling strategies for cloud-augmented clusters or hybrid cloud environments have been explored over the past years. Job submissions can be placed in either the local cluster’s queue or the cloud’s queue depending on a scheduling strategy. Examined in the study are several scheduling strategies that include the shortest queue scheme (to load-balance between the cloud and the cluster), the weighted queue scheme (to redirect all the local requests to the cloud until a pre-defined limit), or the selective backfilling scheme (to give a priority to smaller requests). An online workflow characterization technique based on profiling scientific workflows through collecting fine-grained information like process I/O, runtime, memory usage, and CPU utilization is proposed in [33]. They estimate task runtime, memory consumption and disk space based on input data size of the tasks. They keep dividing and finding correlations between parameters of datasets using clustering techniques. Then the online estimation process MAPE-K monitor and update the system upon information availability. A genetic algorithm can be employed to minimize the job queue time and the cost, when outsourcing queued jobs to the cloud [34]. However, because the genetic algorithm has to examine a workflow and map all the constituent tasks to VMs prior to the execution, complete information about the workload must be available before its execution. A scheduling algorithm for a hybrid cloud environment comprised of failure-prone resources is also investigated [25]. Their goal is to decrease the completion time of the tasks which can be delayed due to resource failures. Because short tasks are less likely to be affected by failures, the proposed algorithm redirects large and long jobs to reliable resources. Our approach is different in that we use a single cloud infrastructure rather than outsourcing overspill tasks to another cloud or a computing cluster.

## Conclusion

This paper presented resource provisioning algorithms for heterogeneous tasks on IaaS clouds. The primary goal of them is to reduce any possible slowdowns while being able to complete task executions within a certain budget. Our algorithm takes into account the available budget when making provisioning decisions and schedules tasks considering the execution time on each VM, significantly improving task performance in an environment comprised of heterogeneous resources. Of particular interest, the CA algorithm minimizes the performance degradation from non-ideal task assignments, reducing the task slowdown rate up to 70%. This underscores the effectiveness of adjusting the allocation of virtual machines of specific type according to input task’s need.

We conclude this paper by suggesting some directions for future work. A key aspect that should be considered when determining the total cost of task executions in a distributed environment is the cost of data transfer from one machine to another. One avenue we want to explore is possible schemes to exploit data locality as well as storage cloud services for resource provisioning. Another missing feature is a means for a user to express desired quality-of-service requirements. For instance, a user may want to minimize execution cost, while prioritizing the execution of particular tasks.

## Supporting Information

S1 File Source code of the proposed algorithm. (RAR)

S2 File Raw results from simulation runs. (XLSX)
